# Tree demography dominates long‐term growth trends inferred from tree rings

**DOI:** 10.1111/gcb.13410

**Published:** 2016-08-16

**Authors:** Roel J. W. Brienen, Manuel Gloor, Guy Ziv

**Affiliations:** ^1^ School of Geography University of Leeds Woodhouse Lane Leeds LS2 9JT UK

**Keywords:** climate change, CO_2_ fertilization, dendrochronology, growth stimulation, population dynamics, sample bias, tropical forests

## Abstract

Understanding responses of forests to increasing CO_2_ and temperature is an important challenge, but no easy task. Tree rings are increasingly used to study such responses. In a recent study, van der Sleen *et al*. (2014) *Nature Geoscience*, 8, 4 used tree rings from 12 tropical tree species and find that despite increases in intrinsic water use efficiency, no growth stimulation is observed. This challenges the idea that increasing CO_2_ would stimulate growth. Unfortunately, tree ring analysis can be plagued by biases, resulting in spurious growth trends. While their study evaluated several biases, it does not account for all. In particular, one bias may have seriously affected their results. Several of the species have recruitment patterns, which are not uniform, but clustered around one specific year. This results in spurious negative growth trends if growth rates are calculated in fixed size classes, as ‘fast‐growing’ trees reach the sampling diameter earlier compared to slow growers and thus fast growth rates tend to have earlier calendar dates. We assessed the effect of this ‘nonuniform age bias’ on observed growth trends and find that van der Sleen's conclusions of a lack of growth stimulation do not hold. Growth trends are – at least partially – driven by underlying recruitment or age distributions. Species with more clustered age distributions show more negative growth trends, and simulations to estimate the effect of species’ age distributions show growth trends close to those observed. Re‐evaluation of the growth data and correction for the bias result in significant positive growth trends of 1–2% per decade for the full period, and 3–7% since 1950. These observations, however, should be taken cautiously as multiple biases affect these trend estimates. In all, our results highlight that tree ring studies of long‐term growth trends can be strongly influenced by biases if demographic processes are not carefully accounted for.

## Introduction

Understanding the response of forests to global change is important as forests are an integral part of the global carbon cycle (Booth *et al*., 2012), taking up more than a quarter of the annual CO_2_ emissions from fossil fuel burning (Pan *et al*., 2011; Le Quéré *et al*., 2013). Forests thus act as an important brake on the rate of CO_2_ increase in the atmosphere and greenhouse warming. The primary cause for the net carbon uptake by forests globally is believed to be a beneficial effect of elevated atmospheric CO_2_ on plant photosynthesis and stomatal conductance (Lloyd & Farquhar, 1996), leading to long‐term growth increases at the stand level (Lewis *et al*., 2009; Phillips *et al*., 2009; Büntgen *et al*., 2014). However, despite the important role of forests for global climate, there is very little information on the magnitude and duration of growth changes in individual trees, and the effect of CO_2_ on *in situ* tree growth remains disputed (see Muller‐Landau, 2009; Clark *et al*., 2010; Wright, 2013).

Growth trends in trees derived from plot studies are often based on relatively short records and are thus of limited use to conclusively determine the drivers behind the observed responses. One seemingly ideal solution is the use of tree rings as they provide information on trees' growth rates over much longer periods and allow extending growth series to preindustrial times. Tree ring records are also relatively easy to collect and ring widths can be measured with great precision. In addition, tree rings allow simultaneous measurements of stable carbon isotopes, providing insights on changes in tree functioning, such as changes in the intrinsic water use efficiency. The greater the plant water use efficiency, the more carbon is fixed per unit water lost, and hence, increases in intrinsic water use efficiency are expected to promote plant growth if water use remains the same (Franks *et al*., 2013). As a result, various recent studies have strongly advocated the use of tree rings to study trees' responses to climate and CO_2_ and to assess the role of forests in the global carbon cycle (Zuidema *et al*., 2013; Babst *et al*., 2014).

In a recent study, Van der Sleen *et al*. (2014) showed that intrinsic water use efficiency (iWUE) derived from tree ring carbon isotopes increased across 12 tropical tree species from three continents in recent decades. Differently from other studies, they have properly accounted for changes in iWUE through tree ontogeny (size and age, see McDowell *et al*., 2011), and thus, this result is more credible than similar results from other studies using carbon isotopes to infer trends in iWUE (Franks *et al*., 2013). A surprising second finding of their study is that increases in iWUE do not result in significant growth increases. Instead, in a more detailed analysis on long‐term growth patterns of these species, they concluded that growth rates mostly decreased over time (Groenendijk *et al*., 2015). This is contrary to expectations and to findings of stand‐level growth increases from monitoring studies in South America and Africa (Lewis *et al*., 2009; Phillips *et al*., 2009; Brienen *et al*., 2015).

**Figure 1:**
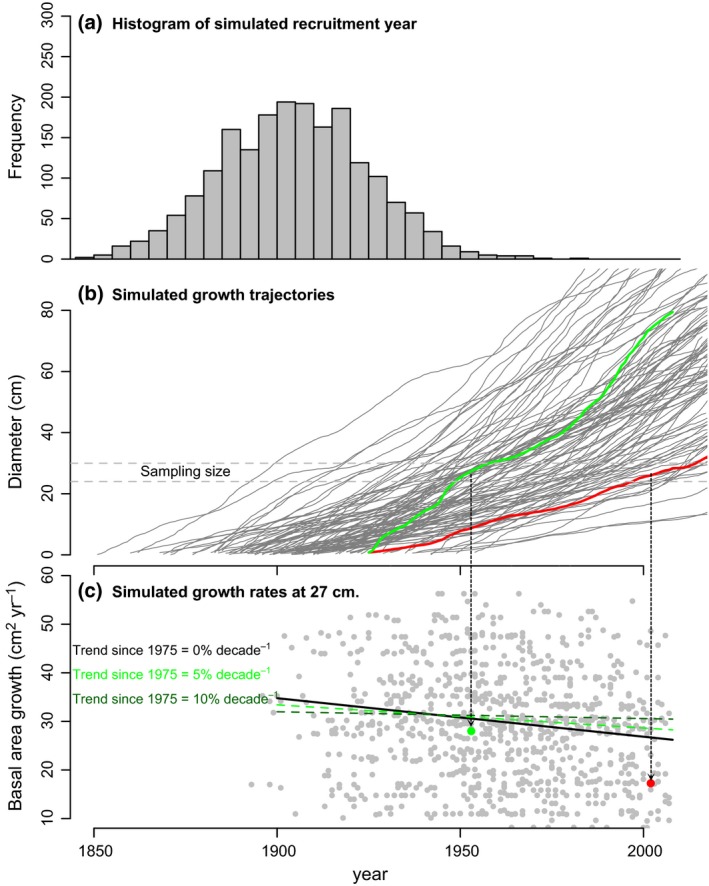
Illustration of the effect of unimodal recruitment on growth trends. (a) Histogram of simulated recruitment year with a unimodal recruitment pattern centred around 1900 (and with standard deviation of 20 years), (b) simulated growth trajectories (from Brienen *et al*., 2012) highlighting in green a fast‐growing tree and in red a slow‐growing tree, both born in 1925 and (c) the effect of unimodal recruitment on growth trends calculated at a fixed size class (‘sampling size’) of 27 cm in diameter (cf. van der Sleen *et al*., 2014) resulting in an apparent negative growth trend (black line) even when growth rates did not change. Note that these trends are calculated from trajectories alive in the ‘sampling’ year of 2010, and by dating the year of ring formation when trees were 27 cm in diameter. Average trend is −2.8% per decade (± 0.42% standard deviation for 500 simulations). The cause for the negative trend is that fast‐growing trees reach the sampling size earlier than slow‐growing trees and thus high growth rates (green dot) tend to be recorded preferentially further back in time compared to slow growth rates (red dot). This negative trend may mask simulated growth increases of 5% or 10% per decade since 1975 (green lines).

The authors evaluate to what extent well‐documented biases in their tree ring data affect their results and conclude that it is very unlikely that these prevented the detection of long‐term growth trends. However, they did not account for the effect of one possible cause for their outcome, which is underlying age distributions clustered around a particular age. Several of their species show little recruitment over recent times (see Vlam, 2014), resulting in an age distribution similar to that shown in Fig. 1a. Such unimodal distributions may result in masking possible growth stimulation if growth rates are calculated in fixed size classes. The reason is simple and can be understood from an extreme case where all trees in the population are born in the same year somewhere in the distant past (red and green lines in Fig. 1b). Persistent growth differences result in fast‐growing individuals reaching the size class in which growth rates are calculated at a younger age compared to slow growers. Therefore, when tracking growth rates back in time, fast growth rates tend to have younger calendar dates when reaching a fixed size compared to slow growth rates. The result is an apparent negative growth trend over time (Fig. 1c). This bias only occurs in approaches such as applied by van der Sleen *et al*. (2014) if growth rates are calculated in a fixed size class. This effect was first described by Vlam (2014), and its possible influence on growth trend evaluations was discussed by Groenendijk *et al*. (2015). However, a formal assessment of the magnitude of this bias on apparent trends in the studies of van der Sleen *et al*. (2014) and Groenendijk *et al*. (2015) has not been performed.

We here examine and quantify the effect of nonuniform recruitment on growth trends presented by van der Sleen and Groenendijk using various approaches. The outline of this article is as follows: we first illustrate the effect of the bias using stochastic simulations of tree ring trajectories (cf. Fig. 1). We then address the question as to what degree observed trends can be explained by uneven age structures. In the last section, we use two approaches to remove the effect of uneven age structures from the data, and re‐evaluate the growth trends observed in their species. The article ends with a discussion of the results and general implications for tree ring studies aiming to detect growth trends.

## How do clustered age distributions affect growth trends?

We first illustrate the magnitude of the effect of a clustered age distribution on historical tree growth reconstructions when calculating growth in a fixed size class as performed by van der Sleen *et al*. (2014) and Groenendijk *et al*. (2015). We do this by sampling simulations of individual growth trajectories based on observed growth data of *Cedrela odorata* from Bolivia (see Brienen *et al*., 2012) in the same way as van der Sleen *et al*. (2014). These growth trajectories contain a realistic autocorrelation structure in time (Brienen *et al*., 2006) resulting in variation in ages among trajectories comparable to the observed variation. The simulated population was centred around 1900 with a standard deviation of 20 years. Full details on this simulation approach can be found in the Text S1.

The outcome of these simulations and the effect of clustered age distributions on apparent growth trends are shown in Fig. 1. We refer to these growth change observations as ‘*apparent trends*’, as the real (or simulated) growth did not change over time and thus there should be no trend observed. It shows that even in the absence of a growth stimulation, clustered age structures result in negative growth trends of 2.8% (± 0.42% SD for 500 simulations) per decade when calculating growth in a fixed size class as performed by van der Sleen *et al*. (2014) and Groenendijk *et al*. (2015). We also tested whether a clustered age distribution could conceal growth stimulations of a similar magnitude as those observed in permanent sample plot studies (i.e. 8% per decade, cf. Brienen *et al*., 2015). To this end, we simulated a (linear) growth increase of 5% and 10% per decade starting in 1975. We specifically chose to apply growth stimulations only over the most recent period, as atmospheric CO_2_ concentrations and plant water use efficiency only increased strongly over recent decades. These simulations show that growth stimulations of 5% or even 10% per decade since 1975 can remain completely undetectable, if ages are clustered as shown in Fig. 1a. This will hold for any tree ring data set that has an underlying age structure that is clustered in time. Thus, it does not matter whether the uneven age structure arises due to limited recruitment, failure to sample smaller and younger trees, use of a minimum sample size limit in the field, or for any other reasons.

The simulations show that the existence of a nonuniform age structure in the tree ring data set suffices to give rise to strong spurious growth rates when calculating growth in a single size class (i.e. the size class isolation method, cf. Peters *et al*., 2015). An alternative standardization approach used by Groenendijk *et al*. (2015) to correct for size‐related trends in tree ring data, the so‐called Regional Curve Standardization (RCS) approach (cf. Briffa *et al*., 1992), results in similar negative apparent growth trends and thus does not remedy the problem. This approach basically uses a size–growth curve of the entire population to standardize growth data (for details, see Appendix).

## Do nonuniform age structures explain reported apparent growth trends?

The data of the species used by van der Sleen *et al*. (2014) show widely varying trends for the different species (see Table 1). van der Sleen *et al*. (2014) evaluated to what degree these trends were affected by known biases described by Brienen *et al*. (2012) and Bowman *et al*. (2013) and show that apparent trends in three of their species, *Melia azedarach*,* Sweetia fruticosa* and *Afzelia xylocarpa,* are affected by negative biases due to mortality effects (e.g. ‘predeath bias’ and ‘juvenile selection effect’, see Groenendijk *et al*., 2015). These biases lead to negative growth trends similar to expectation for the nonuniform age bias, and we therefore exclude these species from the analysis, which evaluates the effects of the nonuniform age bias on overall growth trends.

**Table 1:** Apparent and shuffled growth trends, and age–growth and age–calendar year relationships by species. For details on how shuffled trends were estimated, see main text and Text S1. Note that we excluded from the main analysis the three species that were identified by Groenendijk *et al*. (2015) to have mortality biases, but results for these three species are shown at the bottom of the table in italic. Values in black are significant at *P* < 0.05

Species	Recruitment pattern^a^	Biases^b^	Canopy trees (27 cm)						Understory trees (8 cm)					
			Trends (% per decade)		Age–growth		Age–calendar year		Trends (% per decade)		Age–growth		Age–calendar year	
			Apparent	Shuffled	Pearson's *r*	*P*	Pearson's *r*	*P*	Apparent	Shuffled	Pearson's *r*	*P*	Pearson's *r*	*P*
*Ampelocera ruizii*	Logistic decline		−7.60%	−2.45%	**−0.38**	**0.03**	0.07	0.71	14.55%	−5.12%	**−0.32**	**0.00**	0.07	0.50
*Brachystegia cynometroides*	Unimodal		−6.30%	−4.27%	**−0.40**	**0.00**	**0.65**	**0.00**	0.18%	−2.83%	**−0.33**	**0.00**	**0.24**	**0.01**
*Brachystegia eurycoma*	Unimodal		−0.84%	−1.32%	−0.17	0.15	**0.28**	**0.01**	−3.65%	−2.28%	**−0.34**	**0.00**	**0.52**	**0.00**
*Cariniana ianeirensis*	Logistic decline		2.53%	−0.70%	−0.20	0.13	−0.01	0.93	0.00%	−0.63%	**−0.41**	**0.00**	−0.01	0.95
*Chukrasia tabularis*	Unimodal		1.37%	−1.67%	**−0.27**	**0.05**	**0.43**	**0.00**	−1.94%	−2.33%	−0.14	0.19	**0.25**	**0.02**
*Daniellia ogea*	Unimodal		2.66%	−0.80%	**−0.24**	**0.02**	0.09	0.40	2.80%	0.00%	0.11	0.30	0.06	0.53
*Hura crepitans*	Exponential decline		2.79%	−1.20%	**−0.51**	**0.00**	0.04	0.77	0.42%	−0.62%	**−0.27**	**0.02**	0.06	0.59
*Terminalia ivorensis*	Unimodal^c^		3.37%	−0.62%	**−0.45**	**0.00**	−0.19	0.07	1.92%	−0.52%	**−0.61**	**0.00**	−0.02	0.83
*Toona ciliata*	Unimodal		−0.53%	−0.69%	−0.20	0.17	−0.02	0.87	−14.54%	−0.93%	−0.11	0.45	0.23	0.11
*Melia azedarach*	*Unimodal*	*Predeath* b	*−7.24%*	*−2.41%*	***−0.53***	***0.00***	*0.18*	*0.12*	*−9.16%*	*−2.01%*	***−0.47***	***0.00***	*0.18*	*0.12*
*Sweetia fruticosa*	*Exponential decline*	*Predeath* b	*−7.67%*	*−0.81%*	*−0.17*	*0.28*	*−0.22*	*0.17*	*−1.41%*	*−0.98%*	***−0.35***	***0.00***	*0.05*	*0.64*
*Afzelia xylocarpa*	*Unimodal*	*Juvenile selection* b	*1.00%*	*−0.21%*	***−0.33***	***0.00***	*0.16*	*0.16*	*1.65%*	*−0.85%*	***−0.55***	***0.00***	*0.10*	*0.37*

a Recruitment pattern classification is from Vlam (2014).

b Biases identified by Groenendijk *et al*. (2015), but results for these three species are shown at the bottom of the table in italic. Values in black are significant at *P* < 0.05.

c Rrecruitment pattern of Vlam (2014) differs from the data set used by van der Sleen *et al*. (2014), which consisted of two cohorts.

Data for the three species with biases according to Groenendijk et al. (2015) are in italic, and values in black are significant at *P* < 0.05.

**Figure 2:**
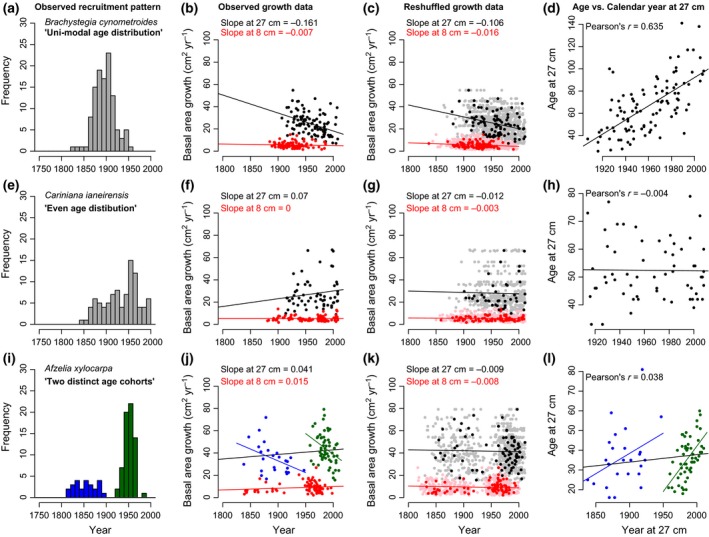
Examples of the effect of age distributions on growth trend observations in three selected species of van der Sleen. Left panels show the age or recruitment patterns, for *Brachystegia* with a unimodel age distribution, *Cariniana* with an even recruitment over time (resulting in a ‘logistic decline’‐type age distribution, cf. Vlam, 2014) and *Afzelia* with two distinct age cohorts (distinguished by blue and green colours). Panels in the second column show the resulting observed growth data and trends over time for two size classes (black points, at 27 cm; red points, at 8 cm in diameter). Panels in third column show the predicted trends due to underlying age distribution using the reshuffling approach (see main text). Panels on the right show the relation between calendar year and age when reaching the sample size of 27 cm. Unimodal age distributions, such as in *Brachystegia* (upper panels), lead in theory to negative growth trends, which are both observed and replicated using the shuffling approach. Such underlying recruitment patterns also result in a close relationship between age and calendar year at sampling size, and strong indication that growth data could be biased.

Several species used in the studies by van der Sleen *et al*. (2014) and Groenendijk *et al*. (2015) exhibit a nonuniform age or recruitment distribution. In Fig. 2, we show examples of the recruitment time distribution for three species along with their apparent growth trends (see Fig. S2 for all 12 species and full names). It is readily apparent that species with a strongly clustered age distribution such as *Brachystegia cynometroides* have a negative growth trend over time, while species with a more uniform age distribution such a *Cariniana ianerensis* do not show a negative trend. Recruitment patterns for some species such as *Afzelia xylocarpa* show two distinct cohorts, leading to strongly negative growth trends for the separate cohorts, while the overall trend is relatively small. Of the 12 species studied, Vlam (2014) concluded that at least 8 have clustered or unimodal recruitment time distributions, while four have relatively uniform age distributions (cf. logistic or exponential decline, see classification in Table 1). While these patterns illustrate the effect of different age distributions on growth trends, it is qualitative in nature and does not prove that age distributions are indeed the cause of these apparent trends.

**Figure 3:**
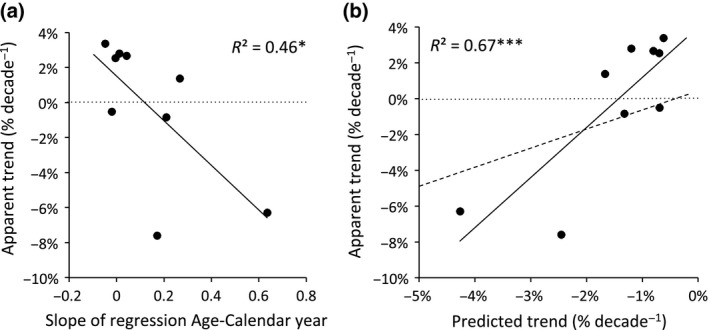
(a) Relationship between apparent growth trends (% per decade) and slopes of the regression between age and calendar year for canopy trees (i.e. at 27 cm diameter) and (b) relationship between apparent and predicted (shuffled) trends. Points falling above the dashed line (1 : 1) in panel (b) suggest positive growth increase for those species. Note that this analysis excluded species with negative biases due to mortality effects (*Afzelia, Melia* and *Sweetia*).

A useful, more quantitative diagnostic of the degree to which age distributions in the data sets are clustered is the relationship between the age at which trees reach the sample size class and calendar year at that size class. For species with continuous regeneration, one would not expect to find a strong relationship between these two variables, whereas in the extreme case, if all trees were born in the same year, this relationship would be perfect (1 : 1). Thus, the strength of the age–calendar year relationship can be used to probe the likelihood for a bias in apparent trend evaluations. This is, of course, only true if the age at which trees reach the sample size class is (negatively) related to the realized growth in that size class. All species show a negative relation between growth and the tree age in that size class (see Table 1), and we therefore expect species with a strong positive relationship between age and calendar year to be negatively biased in their growth trends. The data of van der Sleen *et al*. (2014) do indeed show that species with a strong positive relationship between age and calendar year, such as *Brachystegia cynometroides*, have negative apparent growth trends, while species with more even age structures such as *Cariniana* do not show negative trends (Fig. 1, and Fig. S2). Comparison of apparent trends and the strength of the relationship between age and calendar year across all species shows a negative correlation between the two measures (see Fig. 3a), thus indicating that apparent trends by van der Sleen may – at least partially – be caused by the shape of the underlying age distribution. Note that the three species previously identified by Groenendijk *et al*. (2015) as having negative biases due to mortality effects were omitted from this comparison and that the remaining sample size is thus quite small.

The growth rate over time could be affected by growth stimulation or depression. To isolate just the effect of underlying age distribution on growth trends, we randomly shuffled the observed growth trajectories of the trees in the tree ring data set of van der Sleen *et al*. (2014). This approach consists of randomly initializing trajectories drawing from the observed recruitment time distribution by permutating the start dates of the trajectories. This removes any existing growth stimulation (or depression) trends, for example due to increasing CO_2_ in the data as we start ‘fast‐growing’ and ‘slow‐growing’ trees irrespective of their original birth year while retaining the number distribution of recruits in time. If these shuffled data still show trends (when plotting growth against shuffled calendar date), then those trends must be caused just by the specific age distribution (i.e. number of recruits through time). The exact procedure, and a test for the effectiveness of the procedure to separate real growth changes from changes induced by underlying recruitment patterns, is described in the Text S1.

The time trends of the shuffled growth data for all species are given in Table 1 and in Fig. S1. We find for all species negative slopes, except for *Daniellia ogea* in size class 8. While the simulated negative slopes are for some species relatively small and individually not significant, a two‐tailed *t*‐test shows that the mean of the 500 simulated slopes is significantly different from zero for all species (see Table S1). For most species, the apparent slopes are close to the shuffled slopes, or slightly higher. However, for four species, the apparent slopes were more negative than the shuffled slopes. The two species with the largest differences are *Melia azedarach* and *Sweetia fruticosa,* which were identified previously by Groenendijk *et al*. (2015) to be biased towards lower growth rates over recent times due to elevated mortality at low growth rates (cf. ‘predeath bias’, Bowman *et al*., 2013). As shuffling of the growth data removes this recent negative growth bias, one would for these species indeed expect that the shuffled trends are less negative than apparent trends. When excluding the three biased species (*Afzelia, Melia* and *Sweetia*), we do find a close relationship between the shuffled slopes and the apparent slopes at the sample size of 27 cm in diameter (Fig. 3b). This is consistent with the outcome shown above for age vs. calendar year (Fig. 3a) and suggests that trends for those species that were identified not to have additional biases are likely to be driven by their age (or recruitment) distribution.

It should be noted that the shuffling approach allows estimation of the effect of underlying nonuniform recruitment distribution on growth trends, but does not account for effects of forest dynamics (e.g. thinning or increasing competition) on growth trends. Some of the species such as *Brachystegia cynometroides* have apparent trends that are more negative than estimated by shuffling of the trajectories, which may in fact be due to increasing competition over time for trees after the initial recruitment event. Specifically for the sites in Thailand and Cameroon, the disturbance history indicates that some species have regenerated after high‐intensity, large‐scale disturbances such as fire or windstorms (Vlam, 2014). This may result in greater resources (sunlight, nutrients and water) for those trees that are initially established and less resources for later establishing trees or trees that were initially disadvantaged. This further affects the observed trends by driving down growth trends over time.

## Removing biases

We now want to investigate whether the data of van der Sleen indeed show increased growth or not. To this end, we used three different approaches to remove the trends due to the specific age distributions from effectively occurring trends, for example due to global change, and re‐assess the observed aggregated growth trends for the data set of van der Sleen *et al*. (2014). In the first approach, we correct the original data for the nonuniform age bias by removing for each species the slopes estimated after applying the shuffling method and then recalculate the aggregated overall slope for the nine species using a linear mixed‐effects model approach (see details in the Text S1). In the second approach, we added age as a predictor for growth into the linear mixed‐effects model, which effectively removes the positive relation between age and calendar year at which trees reach sample size arising from the nonuniform age bias (see Fig. 2). In the third approach, we simply leave out those species that have clustered age distributions from the slope estimate using a mixed‐effects model. A test of the effectiveness of the first two approaches to remove the effects of clustered age distributions is provided in the Text S1 and Fig. S3. The outcome of the three different approaches is presented below. Note that to obtain unbiased estimates of long‐term growth trends, we leave out the three species that were identified by Groenendijk *et al*. (2015) to have mortality biases (unrelated to the bias we examine here). This approach differs from analyses by van der Sleen *et al*. (2014) and Groenendijk *et al*. (2015), which included all species, even those with known biases.

### Approach 1: removing of bias using shuffled trends

**Figure 4:**
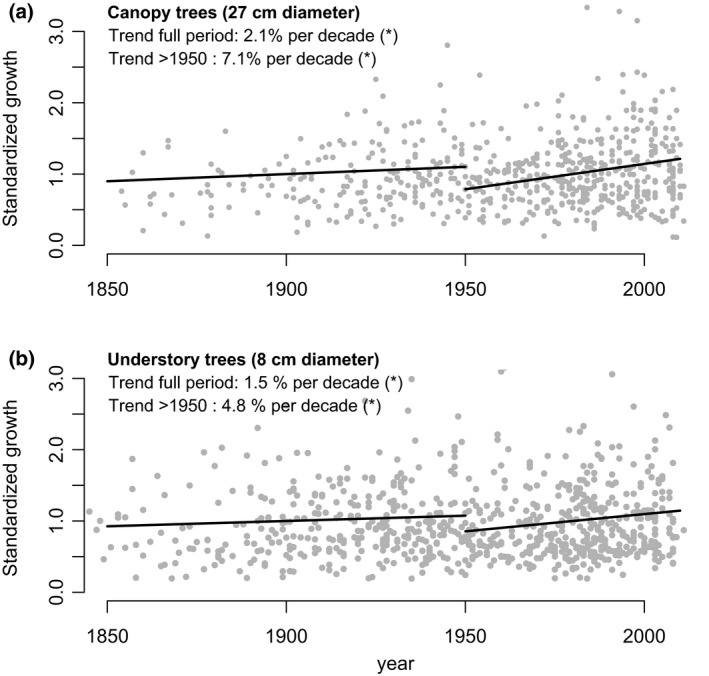
Predicted growth trends after removal of the uneven age bias from the aggregated data set. Panels show trends for the full time period and the most recent period (>1950) for canopy trees (a) and understory trees (b). Shown are standardized growth data, allowing presentation of growth rates for all nine species in one single graph. Aggregated trends are estimated using linear mixed‐effects models from the lme package in r, see Pinheiro *et al*. (2015) with a variance structure as detailed in the Text S1. Note that these trends include only those nine species that are not biased by mortality biases.

The trends from the shuffled growth data provide a baseline for expected trends if growth rates did not change over time (in other words, if there had not been a positive or negative stimulus on growth). If observed trends are lower (more negative) than the shuffled trend, then growth should have decreased over time, whereas if observed growth trends are higher, one would conclude that growth increased over time. Comparison of the shuffled and observed trends for canopy trees shows that for seven of the nine species, the observed trends are larger than the trends based on shuffling and that growth trends are thus actually positive (Table 1). Results for smaller trees are similar with seven out of the nine species showing observed trends larger than the reshuffled trends. To formally test whether removing the age bias does result in significant growth increases, we corrected the original growth data of the nine species for the age bias. We do this for each data point by removing the difference between the predicted growth rate based on the reshuffled trend line, and the simple arithmetic mean growth for that species. Correction according to this procedure leads to trends for each species that are similar to the difference between observed and shuffled slopes, and maintains a data set with similar variation as originally observed (see Fig. S4). We then tested whether the aggregated ‘corrected’ trends for the nine species showed significant changes over time, using the same linear mixed‐effects model as van der Sleen *et al*. (2014). The result of this analysis reveals significant growth increases for the nine species (in the corrected data), while the original trend for the same set of species was not significant (Table 2, Fig. 4). The observed increase is relatively weak when calculating over the full period (i.e. 2.1% and 1.3% per decade for canopy and understory trees, see Table 2), but much stronger when focusing on trends since 1950 (7% and 5% for canopy and understory trees, respectively, see Table S2).

**Table 2:** Results of long‐term trend estimates using linear mixed‐effects model. Models were developed with the lme package (Pinheiro *et al*., 2015) with species as factor with random slope and intercept. The ‘original data’ used the uncorrected growth data for the nine species, the ‘corrected data’ use growth data adjusted for the difference of the shuffled trends from zero (see Text S1), the third model corrects for the nonuniform age distribution by adding age as second explanatory variable, and the last model excluded the three species (*Brachystegia cynometroides, Brachystegia eurycoma* and *Chukrasia tabularis*) with clearly clustered age distributions. Note that all models excluded the three species (*Melia azedarach*,* Sweetia fruticosa* and *Afzelia xylocarpa*) that have negative biases due to mortality effects (see Groenendijk *et al*., 2015). See Text S1 for details and exact model formulation, and Table S2 for the full outcome of various models, Values in black are significant at *P* < 0.05

	Canopy trees			Understory trees			Number of species
	Trends (% per decade)	*P*‐level	AIC	Trends (% per decade)	*P*‐level	AIC	
1. Original data	0.8%	0.491	5194	0.8%	0.356	4489	9
2. Corrected data	**2.1%**	**0.023**	5193	**1.3%**	**0.046**	4492	9
3. Adding age as explanatory variable	**1.6%**	**0.040**	5164	1.0%	0.105	4430	9
4. Excluding species with age bias	**2.3%**	**0.008**	3373	**1.3%**	**0.036**	3022	6

Values in bold are significant at *P* < 0.05.

### Approach 2: adding age to linear mixed‐effects model

A second approach to correct for the nonuniform age distribution bias and formally test for growth changes over time involves using a statistical model to remove the age effect on growth. This can be achieved simply by adding age (at the moment that a tree reaches sampling size), as a second explanatory variable to the linear mixed‐effects model, which effectively removes the nonuniform age bias (see Fig. S3). The outcome of this analysis shows that growth in canopy trees increased by 1.6% for the full period and 6% per decade since 1950. Increases in understory trees are slightly lower for the full period, (see Table 2 and Table S2).

### Approach 3: excluding species with nonuniform age distributions

Finally, we evaluated the aggregated growth trends by leaving out all species with biases. Thus, apart from the three species already excluded due to identified mortality biases (see Groenendijk *et al*. (2015)), we also excluded species with strongly nonuniform age distributions. *Brachystegia cynometroides, Brachystegia eurycoma* and *Chukrasia tabularis* all have peaked age distributions (see Fig. S2) and show significantly positive relationships between age and calendar year at both 8 cm and 27 cm (see Table 1). There is thus strong reason to assume that these three species are biased by the nonuniform age distribution bias. Excluding these from the analysis results in significant growth increases for the remaining six species of 2.3% and 1.3% per decade for canopy and understory trees (see Table 2 and Table S2).

## Discussion

van der Sleen *et al*. (2014) and Groenendijk *et al*. (2015) carefully evaluated possible biases that may have affected their estimates of growth trends. However, despite their assessment, we find that the observed trends in the data set of van der Sleen *et al*. (2014) are still affected by biases. Firstly, they did not account for mortality biases due to loss or removal of slow‐growing trees from the data set further back in time (e.g. ‘predeath bias’, Bowman *et al*. (2013) and ‘juvenile selection effect’, Rozendaal *et al*. (2010)). In their assessment, Groenendijk *et al*. (2015) conclude that three species are likely affected by these mortality biases, but these species were not removed from their analysis. Removal of the species from the analysis resulted in trends that are more positive compared to the full data set including the biased species. Secondly, they did not correct for the bias caused by nonuniform age distributions. Our analyses show that age distributions affect the apparent observed trends. Species with more clustered age distributions – as diagnosed, for example by its age vs. calendar year relationship (see Table 1) – have more negative slopes (see Fig. 3a). In addition, the slopes obtained by the shuffling approach, which are exclusively due to the underlying age distribution, are closely related to the apparent growth trends (Fig. 3b). We therefore conclude that variation in trends between different species of the van der Sleen *et al*. (2014) data set is an artefact. We could not verify whether this holds for the analysis performed by Groenendijk *et al*. (2015) using data from across all size classes of the same species, but the effects of biases may vary across size classes. For instance, simulations with tree ring trajectories (similar to those in Fig. 1) showed that spurious positive trends, due to the slow‐grower survivorship bias (see Brienen *et al*., 2012), were more prominent in growth reconstructions at larger size classes (results not shown). We also checked whether the alternative standardization approach (i.e. the adapted Regional Curve Standardization, see Briffa *et al*., 1992) used in the analysis by Groenendijk *et al*. (2015) would result in a similar bias and find that it does (see Fig. S1).

We used three approaches to correct for the nonuniform underlying age distributions and find very consistent results across the different approaches. For all three approaches, we find positive growth increases after correction for the nonuniform age distribution bias. The approaches not only give a similar sign in the trend predictions, but also converge on the magnitude of the growth increase. Estimated growth increases are between 1.1% and 1.5% per decade for understory and between 1.6% and 2.3% per decade for canopy trees using the full time period. When focusing on the period since 1950 when intrinsic water use efficiency increases are strongest (van der Sleen *et al*., 2014), we find that growth increased between 2.9% and 4.85% per decade for understory trees and between 5.3% and 7.3% per decade for canopy trees. This shows that nonuniform age distributions and mortality biases masked growth increases in the data set of van der Sleen *et al*. (2014). Our analysis of their data thus contradicts earlier conclusions that there is no evidence for growth stimulation, for example via CO_2_ fertilization (van der Sleen *et al*., 2014), or that growth would have deteriorated over recent times (Groenendijk *et al*., 2015). However, while growth rate increases are consistent with a CO_2_ fertilization effect on growth and observation from permanent plot monitoring in the tropics (Lewis *et al*., 2009; Brienen *et al*., 2015), it should be noted that other biases may still be causing these apparent growth increases. In particular, positive growth trends may be the result of the slow‐grower survivorship bias (Brienen *et al*., 2012). This bias arises due to differences in longevity between fast‐ and slow‐growing trees with fast growers maturing and dying faster (Black *et al*., 2008; Bigler & Veblen, 2009; Di Filippo *et al*., 2015). The result of this is a spurious lack of fast growth rates over earlier parts of tree ring records. The data set of van der Sleen *et al*. (2014) seems indeed to indicate some lack of fast growth rates at earlier times of the record (see Fig. 4) consistent with this bias. However, it should be noted that the observed growth increases are strongest over recent times, a period that should be less affected by this particular bias (Brienen *et al*., 2012).

Various recent studies find that growth responses derived from tree rings are negative, despite increases in intrinsic water use efficiency (Silva *et al*., 2010; Andreu‐Hayles *et al*., 2011; Penuelas *et al*., 2011). The bias identified here caused by nonuniform age structures could have affected these studies, as large‐scale stand‐replacing disturbances due to wind, fire or large‐scale insect attacks are more common in boreal and temperate forests (Johnson, 1996; Frelich, 2002) than in tropical forests (Espirito‐Santo *et al*., 2014). However, the direction and magnitude of biases depends also on the specific detrending techniques (Briffa & Melvin, 2011; Brienen *et al*., 2012; Peters *et al*., 2015), making it more difficult to actually evaluate and compare the outcome of different tree ring studies.

While tree ring studies are increasingly being advocated as a tool to evaluate long‐term tree growth responses to global change (Zuidema *et al*., 2013; Babst *et al*., 2014), our analysis here provides a stark warning about the suitability of this approach to infer long‐term growth responses. Multiple biases causing positive and negative growth changes affect trend estimates (Cherubini *et al*., 1998; Briffa & Melvin, 2011; Brienen *et al*., 2012; Bowman *et al*., 2013; Nehrbass‐Ahles *et al*., 2014; Vlam, 2014; Peters *et al*., 2015). The effect of some biases can be estimated, and corrected for, as we have shown here for the nonuniform age bias, or can be avoided by carefully designed sample strategies (e.g. ‘Big tree sample bias’, Brienen *et al*., 2012; Nehrbass‐Ahles *et al*., 2014). However, disentangling positive and negative biases may prove more challenging. In addition, biases due to differences in mortality rates and tree longevity for fast‐ and slow‐growing trees are very difficult to account for. These types of biases can be corrected for if dead trees can be sampled and their full growth history reconstructed. For instance, tree ring analysis on dead trees has revealed relationships between tree growth and longevity (Bigler & Veblen, 2009), and growth and mortality rates (Kobe *et al*., 1995). Alternatively, one can use mortality functions from monitoring studies to simulate the effect of mortality on growth trends of surviving trees (see Foster *et al*., 2014). However, both approaches require high confidence in mortality–growth and mortality–age relationships. Without well‐defined growth–mortality relationships, inferences of long‐term growth changes from tree rings will unfortunately remain very uncertain, especially when using species with relatively short life spans as those are more prone to demographic biases. Thus, while tree rings are very useful for a range of studies, we recommend that the study design and analyses be specifically tailored to account for the potential of each species and site to exhibit bias due to demographic shifts, or lack thereof before tree ring data be used for long‐term growth trend assessment.

## Conclusions

Growth trends observed by van der Sleen *et al*. (2014) are affected by several biases. One particular bias, the nonuniform age bias, has masked historical growth increases. However, even these increases may still be spurious as other biases may be involved as well. We conclude that the results of van der Sleen *et al*. (2014) cannot be used to determine whether CO_2_ fertilization has led to growth increases or not. More generally, most tree ring studies are likely not suitable for evaluation of growth responses to global change, as disentangling effects of (opposing) biases are very difficult.

## Supporting information


**Text S1.** Simulation approach to illustrate effect of clustered distribution on growth trends.
**Table S1.** Results of observed and shuffled trends for canopy and understory trees for all 12 species of van der Sleen *et al*. (2014).
**Table S2.** Outcome of aggregated growth trends estimated using linear mixedeffects models.
**Figure S1.** Effect of Region Curve Standardisation (RCS, cf. Briffa *et al*., 1992) on growth trends under a unimodal age distribution.
**Figure S2.** Observed recruitment patterns, and observed and predicted growth trends at 8 and 27 cm diameter, and age –calendar year relationships for all 12 species of van der Sleen *et al*. (2014).
**Figure S3.** Results of tests of two different correction methods for uneven population structures.
**Figure S4.** Outcome of the reshuffling correction of growth data for each of the species at 27 cm and 8 cm.
