# Supplementary material for: Tree demography dominates long‐term growth trends inferred from tree rings
Source: Glob Chang Biol. 2016 Aug 16;23(2):474–84. doi: 10.1111/gcb.13410 (PMC6849721; doi:10.1111/gcb.13410)
Supplement: Supplementary file 1 — Text S1. Simulation approach to illustrate effect of clustered distribution on growth trends. Table S1. Results of observed and shuffled trends for canopy and understory trees for all 12 species of van der Sleen et al. (2014). Table S2. Outcome of aggregated growth trends estimated using linear mixedeffects models. Figure S1. Effect of Region Curve Standardisation (RCS, cf. Briffa et al., 1992) on growth trends under a unimodal age distribution. Figure S2. Observed recruitment patterns, and observed and predicted growth trends at 8 and 27 cm diameter, and age –calendar year relationships for all 12 species of van der Sleen et al. (2014). Figure S3. Results of tests of two different correction methods for uneven population structures. Figure S4. Outcome of the reshuffling correction of growth data for each of the species at 27 cm and 8 cm. [file GCB-23-474-s001.pdf]

Supporting information

Tree demography dominates long-term growth trends inferred from tree rings.

Roel J.W. Brienen[1], Manuel Gloor[1], Guy Ziv[1].

[1]School of Geography, University of Leeds, Woodhouse Lane, LS2 9JT, Leeds, UK
E-mail for correspondence: r.brienen@leeds.ac.uk, telephone: ++ 44 113 343 3381

Content:

1. Simulation approach to illustrate effect of clustered distribution on growth trends

2. Shuffling procedure.

3. Bias correction approaches

4. Testing the bias correction approaches

SI References

SI Table 1. Results of observed and shuffled trends for canopy and understory trees for all 12 species of Van der Sleen et al. (2014).

SI Table 2 Outcome of aggregated growth trends estimated using linear mixed-effects models

SI Fig. 1 Effect of Region Curve Standardisation (RCS, cf. Briffa et al. 1992) on growth trends under a unimodal age distribution.

SI Fig. 2 Observed recruitment patterns, and observed and predicted growth trends at 8 and 27 cm diameter, and age –calendar year relationships for all 12 species of Van der Sleen et al. (2014).

SI Figure 3 Results of tests of two different correction methods for uneven population structures.

SI Fig. 4 Outcome of the reshuffling correction of growth data for each of the species at 27 cm and 8 cm.

1. Simulation approach to illustrate effect of clustered distribution on growth trends

To illustrate the effect of clustered age distributions on growth reconstructions from tree ring data, we used simulations of individual growth trajectories based on observed growth data of *Cedrela odorata* from Bolivia (see Brienen *et al.*, 2012). These growth trajectories retain a realistic autocorrelation structure of growth over time (Brienen *et al.*, 2006) resulting in variation in ages among trajectories comparable to the observed variation. We use a mortality scenario that resulted in a population size distribution over time similar to the observed distribution (specifically, a constant mortality of 1.5% per year which increases for trees bigger than 100 cm in diameter by 0.1% per year, for details see Brienen et al. 2012). To create a population sample with age distribution clustered around a single age, we randomly drew recruitment (or birth) years for 10.000 individual tree growth trajectories from a normal probability distribution around a central year of 1900 and with a standard deviation of 20 years. To demonstrate the effect of this non-uniform recruitment on long-term growth trend reconstructions, we sampled from these simulated growth trajectories those trees that were still alive in 2011. To probe whether increasing growth over time are still detectable under non-uniform recruitment distributions, we also simulated growth trajectories with linearly increasing growth of 5 and 10% per decade since 1975.

In line with the “size class isolation” approach used by Van der Sleen et al. (2014), we plotted the basal area growth rates of (alive) trajectories at a diameter of 27 cm (taking the average of 5 ring-widths) against the year in which those rings were formed. Groenendijk et al. (2015) also used the Regional Curve Standardisation (RCS) approach (cf. Briffa et al. 1992) to evaluate growth trends. This approach consists of developing an average growth curve for the full dataset, which is then used to detrend the data. Usually this is done using age as a predictor for growth, but Groenendijk et al. (2015) adapted this to use size to standardize the data. We replicated exactly their approach using the data from the simulated population with the non-uniform recruitment distribution. SI Fig. 2 shows the outcome of this analysis. Results are very similar to those with the size class isolation approach, with non-uniform recruitment structure leading to apparent growth decreases of 2.5-3.5% per decade, even when growth did not change.

All analyses were done in R (version 3.1.0), while growth trajectories from Brienen et al (2012) were originally simulated in Matlab and then loaded into R for this analysis.

2. Shuffling procedure.

To assess the strength of non-uniform age distribution on the observed (or apparent) growth trends, we used the following approach. We randomly shuffled the years in which trees recruited by giving each tree a new random recruitment year according to the observed historical recruitment pattern of the population. We then calculated the year at which the tree reaches the sample sizes (8 and 27 cm diameter respectively), by adding its age at 8 and 27 cm to the shuffled recruitment year. This shuffling procedure randomizes growth trajectories between trees only with respect to the year in which the trees were originally born, while maintaining for each species the same historical

recruitment distribution (in other words an identical set of recruitment years). Note that this procedure also maintains the original data-structure in the sense that it uses the same growth rate data with the exact same age and growth rate variation, and maintains the existing negative relation between age and growth rate at sample size (see Table 1). Maintaining this exact data-structure is crucial as all aspects affect the strength of the predicted growth trends. In particular, a negative relationship between the age of a tree at which it reaches the sample size class and its growth rate means that trees that quickly reach the sample size also have a relative high basal area growth at that diameter, which is the main cause for negative trends to arise under unimodal recruitment patterns. To obtain robust estimates of long-term trends, we repeated the reshuffling procedure 500 times and each time calculated the average estimated growth trends in size classes of 8 and 27 cm. Trend estimates were calculated separately for each species as the slopes of an ordinary linear regression. All reshuffled growth observations falling after 2011, the year of sampling, were omitted from the calculation of the slope. The results were moderately affected by this omission, and yielded in all cases more negative slopes when leaving the data after 2011 in the dataset. Data on growth rates (Basal area increment) and year of tree ring formation for size classes 8 and 27 cm were obtained from supplemental info of Van der Sleen et al. (2014), while age data for trees were kindly provided by the authors.

3. Bias correction approaches

We used two approaches to "correct" for the non-uniform age bias. In the first approach we used the predicted growth trend from the shuffling procedure for each species to remove the effect of non-uniform age distributions. This was done by subtracting for each growth point the difference between the simulated (shuffled) trends and the mean growth (slope zero) for that species. Original and corrected growth data and their trends for understory and canopy trees are shown in SI Fig. 4. The resulting growth trends in the corrected data are similar to the slopes estimated as the differences of the shuffled and the apparent trends given in SI Table 1. We then used the corrected growth data to test whether the full dataset of species show aggregated growth trends by replicating the statistical procedure applied by Van der Sleen et al. (2014). This procedure consisted of using a linear mixed-effects model from the R-package lme (Pinheiro *et al.*, 2007) where species are added as factor with random slopes and intercept. In the original analysis Van der Sleen et al. (2014) used log-transformed basal area growth data to obtain homogeneity in the variance of the residuals of the model. We tested models with the same log-transformation. However, for the main analysis we used an alternative, which is the explicit modelling of heterogeneity in the covariates of the dataset. This accounts for the differences in spread of growth rates (basal area increment) between species. The lme package allows testing for a different combination of variance structures with the varComb function. We selected the optimal model using the AIC following Zuur et al. (2009). The optimal model with the lowest AIC for most models had a variance structure allowing unequal variance between species (varIdent(form= ~ 1|species)). The model outcome was similar in sign to that using log-transformed data, however significance levels of the slopes varied in some cases (see SI Table 2). Specifically, in the analysis for the understory size class log-

transformed data did not give significant slopes. Disadvantage of transformation is that one changes the type of relationship, which may leave changes at smaller basal area increment undetectable. It is not directly clear whether transformation is preferable to using raw data, but various authors argue that use of raw data with extended models for heterogeneity, as we do, is usually preferable (Keele, 2008; Zuur *et al.*, 2009; Stroup, 2015). Visual inspection shows that the residuals of the models with raw data and log-transformed data are both approximately homogeneous.

The second approach consists of using the original growth data, but adding the age at which the tree reaches the sample size class to the linear mixed effects model as additional explanatory variable, with variable slope and intercept. As ages differed largely between species, we used in the mixed effects model the difference between a trees' age at sample size and the average age at which that particular species reaches sample size.

A third approach to test for growth trends after removing the non-uniform age bias is simply excluding those species that had strongly clustered age distributions. One good diagnostic for the clustering of age distributions, and thus the likelihood of bias, is the strength of the relationship between age and calendar year. For species with continuous regeneration one would not expect to find a strong relationship between these variables, whereas if all trees were born in the same year, this relationship would be perfect (1:1). We excluded the three species, *Brachystegia cynometroides, Brachystegia eurycoma*, and *Chukrasia tabularis*, which all have peaked age distributions (see SI Fig. 2), and show significantly positive relationships between age and calendar year at both 8 cm and 27 cm (see Table 1). These species are also independently classified by Vlam (2014) to have uni-model recruitment patterns.

<u>4. Testing the bias correction approaches</u>

We tested whether the two methods to correct for the un-even age bias (by reshuffling trajectories or adding age to the mixed effect model) do correctly remove trends due to un-even age structures. To this end, we used the age structure shown in Fig. 1, which is centred around 1900 and has a standard deviation of 20 years. We simulated three scenarios: i) no growth stimulation, ii) a 1% growth stimulation since 1900, and iii) a 5% growth stimulation since 1950. We randomly generated 500 times a population initialized with 10.000 trees, and recalculated each time the predicted growth change according to the two bias correction methods. Note that in the reshuffling procedure we used the average of 500 reshuffles (for each simulation) to replicate exactly the procedure used for the main data analysis. Outcome of the resulting growth trend predictions using the two approaches is shown in SI Fig. 3. It shows that the mean predictions are reasonably close to the forced growth trend. However, the correction method seemingly overestimates the growth trends, especially for the age correction method, even when there is no growth change. This, however, is not an artefact of our correction procedure, but due to a known bias in tree ring data arising as a result of differences in survival chances of slow and fast growing trees (the "slow grower survivorship bias", see Brienen et al 2012). This bias will nearly always lead to apparent positive trends in growth, and is described in detail by Brienen et al. (2012). Even simulations without growth change and regular, even regeneration structure resulted in a positive growth

trend of 0.68% per decade, consistent with the observed (corrected) trend for the age correction (see SI Fig. 3b). The reshuffling method seems to slightly underestimate long-term trends. Note that the large variation between simulation runs was also found between non-corrected slopes, and is thus not due to inaccurate bias correction, but caused by large stochastic variation between individual runs in sampled trajectories and recruitment or age structures. Overall these tests validate the approach used to correct for the un-even age bias.

SI Table 1. Results of observed and shuffled trends for canopy and understory trees for all 12 species of Van der Sleen et al. (2014). Trend slopes are calculated using an ordinary regression. Standard error (SE) is given for the observed slopes. For the shuffled slope the mean of 500 reshuffles is given with the 95% confidence interval using a t-test. T-test indicates that in all species the shuffled slopes are significantly different from the observed slopes. Recruitment patterns are from Vlam (2014) and the possible biases in species were identified in Groenendijk et al. (2015).

	Canopy trees (27 cm)					Understory trees (8 cm)						
**Species**	**Observed slope**	**SE**	**Shuffled slope**	**95% CI (t-test)**	**Difference slope**	**Observed slope**	**SE**	**Shuffled slope**	**95% CI (t-test)**	**Difference slope**	**Recruitment pattern**	**Observations**
Afzelia	0.041	0.03	-0.0087	(-0.0116,-0.0058)	0.0497	0.015	0.01	-0.0077	(-0.0087,-0.0067)	0.0227	Unimodal	Juvenile selection bias
Ampelocera	-0.1903	0.39	-0.0613	(-0.0798,-0.0428)	-0.129	0.0861	0.02	-0.0303	(-0.0323,-0.0283)	0.1164	Logistic decline	
Brachystegia	-0.1614	0.03	-0.105	(-0.1071,-0.103)	-0.0564	-0.0072	0.01	-0.0156	(-0.0164,-0.0149)	0.0084	Unimodal	
Brachystegia	-0.0301	0.06	-0.047	(-0.0514,-0.0427)	0.0169	-0.0188	0.00	-0.0118	(-0.0124,-0.0112)	-0.007	Unimodal	
Cariniana	0.0696	0.05	-0.0193	(-0.0249,-0.0137)	0.0889	-0.0001	0.00	-0.0033	(-0.0039,-0.0027)	0.0032	Logistic decline	
Chukrasia	0.0376	0.06	-0.0499	(-0.0554,-0.0443)	0.0875	-0.0057	0.01	-0.0132	(-0.0147,-0.0118)	0.0075	Unimodal	
Daniellia ogea	0.071	0.03	-0.0214	(-0.0243,-0.0185)	0.0924	0.0131	0.00	0	(-0.0007,0.0007)	0.0131	Unimodal	
Hura crepitans	0.1048	0.08	-0.0451	(-0.0524,-0.0378)	0.1499	0.0059	0.03	-0.0089	(-0.0119,-0.0059)	0.0148	Exponential decline	
Melia	-0.5179	0.16	-0.1725	(-0.1877,-0.1573)	-0.3454	-0.3302	0.13	-0.0724	(-0.085,-0.0598)	-0.2578	Unimodal	Pre-death bias
Sweetia	-0.153	0.04	-0.0161	(-0.021,-0.0112)	-0.1369	-0.006	0.00	-0.0042	(-0.0048,-0.0036)	-0.0018	Exponential decline	Pre-death bias
Terminalia	0.2635	0.08	-0.0488	(-0.0565,-0.0411)	0.3123	0.0427	0.03	-0.0117	(-0.0145,-0.0088)	0.0544	Unimodal	
Toona ciliata	-0.0203	0.11	-0.0264	(-0.0339,-0.0188)	0.0061	-0.1449	0.05	-0.0093	(-0.0146,-0.0039)	-0.1356	Unimodal	

SI Table 2 Outcome of aggregated growth trends estimated using linear mixed-effects models (lme-package, see Pinhero et al. (2007) with species as factor with random slope and intercept. Different models, datasets and species combinations were tested. First, using the original data for all 12 species and excluding the three species biased by mortality effects (*Melia azedarach*, *Sweetia fructicosa* and *Afzelia xylocarpa*). Secondly, using the same sets of species, but with the data that are corrected for the non-uniform age distribution bias (see methods). In the third approach we used the original data, but corrected for non-uniform age distribution bias by adding age as additional explanatory variable to the linear mixed-effects models. And the last model uses the original data, but excluding those species with clearly clustered age distributions (i.e., *Brachystegia cynometroides, Brachystegia eurycoma*, and *Chukrasia tabularis*, see text). Models were run both for the full time period and including only data since 1950. Outcome of significance levels of the slopes for model with log-transformed the data (as used by Van der Sleen et al. 2014) is also shown.

		**Canopy trees (27 cm)**						**Understory trees (8 cm)**					
**Model**	**Data set**	**Mean slope**	**% change**	**P level**	**P-level Log model**	**t-value**	**AIC**	**Mean slope**	**% change**	**P level**	**P-level Log model**	**t-value**	**AIC**
1. Original data	All species (12)	0.0051	0.1%	0.88	0.33	0.1417	6938.989	0.0091	0.91%	0.19	0.56	1.3115	6097.326
1. Original data	Excl. biased species (9)	0.0303	0.8%	0.49	0.95	0.689	5193.922	0.0072	0.86%	0.35	0.84	0.9245	4489.185
2. Corrected data	All species (12)	0.0465	1.2%	0.16	0.70	1.4145	6932.735	**0.0143**	**1.43%**	**0.02**	0.47	2.358	6097.589
2. Corrected data	Excl. biased species (9)	**0.0769**	**2.1%**	**0.02**	**0.02**	2.2431	5192.441	**0.0124**	**1.49%**	**0.05**	0.32	1.9434	4491.784
3. Original data including age	All species (12)	-0.0012	0.0%	0.97	0.52	-0.0327	6878.717	**0.0131**	**1.31%**	**0.04**	0.86	2.0841	5987.342
3. Original data including age	Excl. biased species (9)	**0.0586**	**1.6%**	**0.04**	*0.06*	2.0518	5164.477	0.0094	1.13%	0.10	0.62	1.6299	4432.052
4. Original data 6 species	Excl. all biased species (6)	**0.0996**	**2.3%**	**0.01**	**0.01**	2.6514	3373.294	**0.0139**	**1.37%**	**0.03**	0.49	0.6777	822.3621
**Trend over recent times (>1950)**													
1. Original data	All species (12)	-0.0347	-0.8%	0.76	0.44	-0.311	4916.459	-0.0064	-0.54%	0.55	0.44	-0.6014	3841.134
1. Original data	Excl. biased species (9)	0.2221	6.0%	0.09	0.31	1.6976	3482.369	0.0359	4.01%	0.06	0.75	1.8657	2440.571
2. Corrected data	All species (12)	0.0166	0.4%	0.87	0.75	0.1514	4915.559	0.0051	0.43%	0.63	0.92	0.4834	3843.819
2. Corrected data	Excl. biased species (9)	**0.2629**	**7.1%**	**0.04**	*0.09*	2.0293	3482.07	**0.0428**	**4.78%**	**0.01**	0.28	2.5852	2443.23
3. Original data including age	All species (12)	0.0038	0.1%	0.96	0.64	0.0407	4881.735	**na[1]**	**na[1]**	**na[1]**		**na[1]**	**na[1]**
3. Original data including age	Excl. biased species (9)	0.2411	6.5%	0.06	0.17	1.8971	3465.206	**0.0261**	**2.92%**	**0.03**	0.60	2.1598	2412.145
4. Original data 6 species	Excl. all biased species (6)	0.2303	5.3%	0.19	0.33	1.3252	2265.914	**0.0532**	**5.1%**	**0.00**	0.09	3.4012	1791.339

1) Model is unstable, and does not converge for this dataset.

SI References

Brienen, R.J.W., Zuidema, P.A. & During, H.J. (2006) Autocorrelated growth of tropical forest trees: Unraveling patterns and quantifying consequences. *Forest Ecology and Management*, **237**, 179-190.

Brienen, R.J.W., Gloor, E. & Zuidema, P.A. (2012) Detecting evidence for CO_2 fertilization from tree ring studies: The potential role of sampling biases. *Global Biogeochem. Cycles*, **26**, GB1025.

Keele, L.J. (2008) *Semiparametric regression for the social sciences*. John Wiley & Sons.

Pinheiro, J., Bates, D., DebRoy, S. & Sarkar, D. (2007) Linear and nonlinear mixed effects models. *R package version*, **3**, 57.

Stroup, W.W. (2015) Rethinking the analysis of non-normal data in plant and soil science. *Agronomy Journal*, **107**, 811-827.

Wood, S. (2011) gamm4: Generalized additive mixed models using mgcv and lme4. *R package version 0.1–2*,

Zuur, A., Ieno, E., Walker, N., Saveliev, A. & Smith, G. (2009) Mixed effects models and extensions in ecology with R; Gail M, Krickeberg K, Samet JM, Tsiatis A, Wong W, editors. *New York, NY: Spring Science and Business Media*,

Supporting information Figures

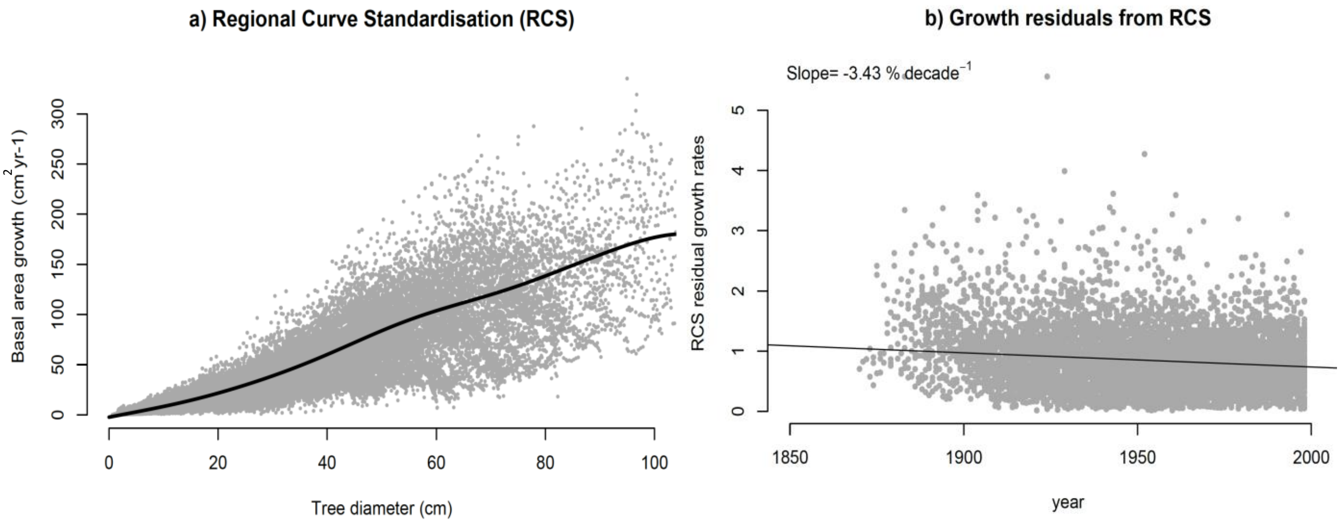

SI Fig. 1 Effect of Region Curve Standardisation (RCS, cf. Briffa et al. 1992) on growth trends under a unimodal age distribution. Panel a) shows basal area growth plotted against tree diameter and the resulting mean diameter-growth curve (or Region Curve) that was used to remove the size effect from the growth data. We used a General Additive Mixed Model (GAMM) from the gamm4 R package (Wood, 2011) to predict the diameter growth curve. Panel b) shows the residuals of the diameter growth curve for the plotted against calendar year for a population with a unimodel age distribution centered around 1900 and with a standar deviation of 20 years, as shown in main article Figure 1. Residuals were obtained by dividing each growth points by the predicted mean diameter growth. The full procedure as described by Groenendijk et al. (2015) was followed.

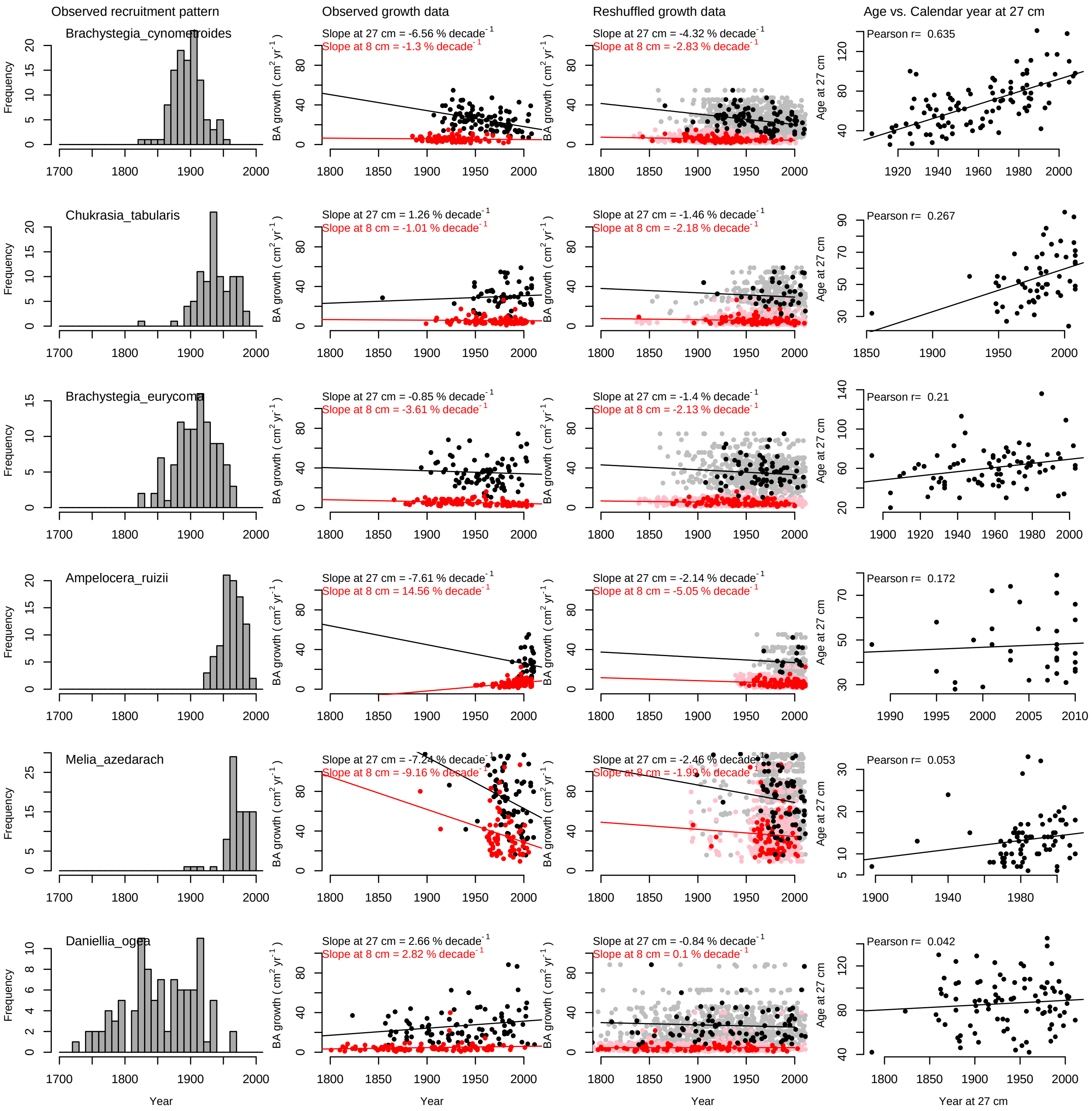

SI Fig. 2 Observed recruitment patterns, and observed and predicted growth trends at 8 and 27 cm diameter, and age –calendar year relationships for all 12 species of Van der Sleen et al. (2014). The left panels illustrate the observed age or recruitment patterns of individual species (y-axis is number of trees), the second column of panels shows the observed basal area growth data (in cm^2 yr^{-1}) at 8 and 27 cm in diameter (red and black dots) and the observed linear trends (red and black lines). Third column of panels illustrate the result of the shuffling of growth data at 8 and 27 cm in diameter (red and black dots) for one shuffle, and for 10 shuffles (pink and grey dots, see SI text for details on shuffling method). Lines indicate the average predicted long-term trends of 500 simulations. Note that trends arising in the reshuffled growth data

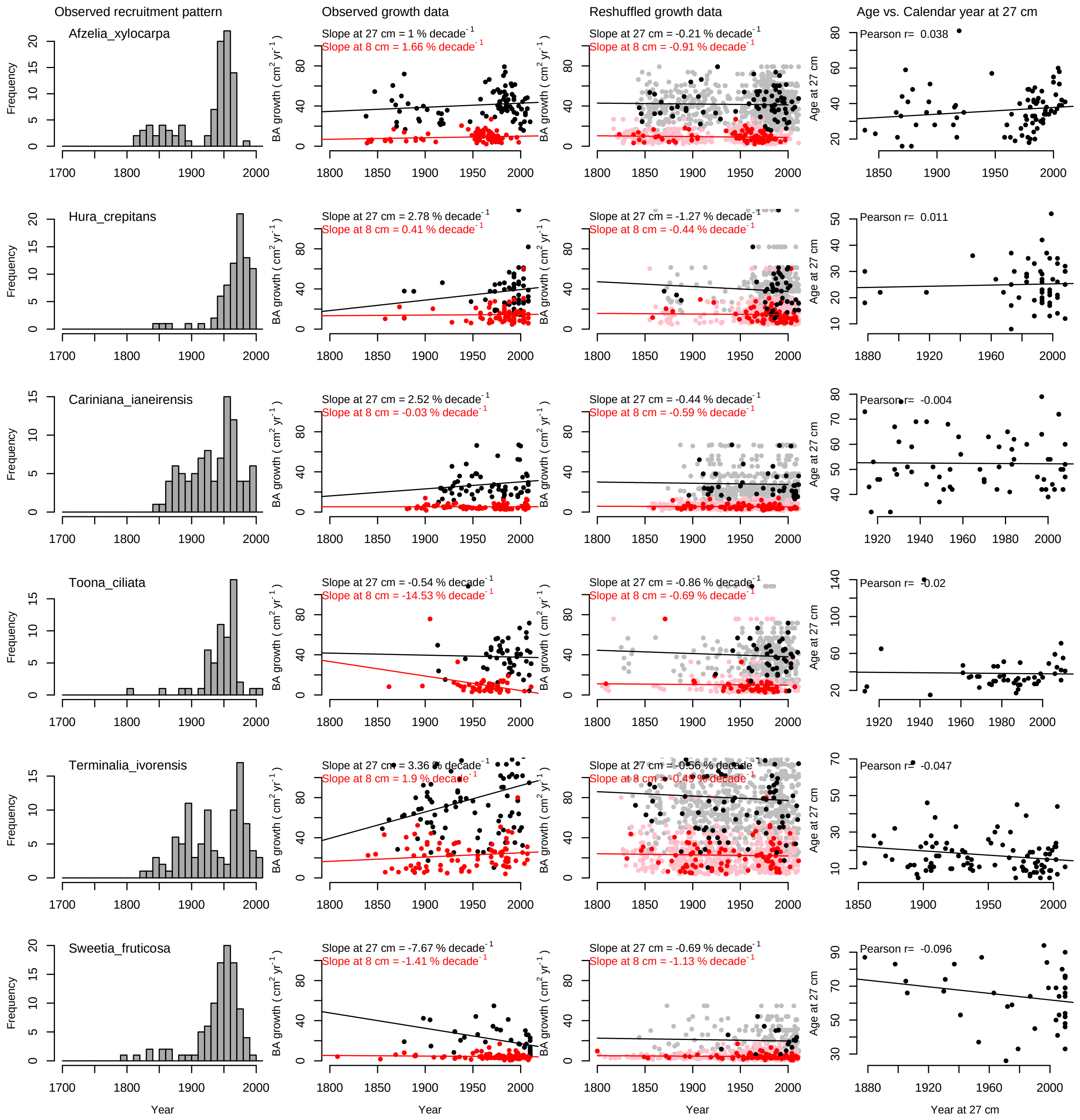

SI Fig. 2 (continued) .. are entirely caused by irregular underlying recruitment patterns of the species as reshuffling removes any time trends. The right panels show the relation between calendar year and age when reaching the sample size of 27 cm. Unimodal age distributions are expected to have close positive relationships between age and calendar year, indicating that growth data could be biased.

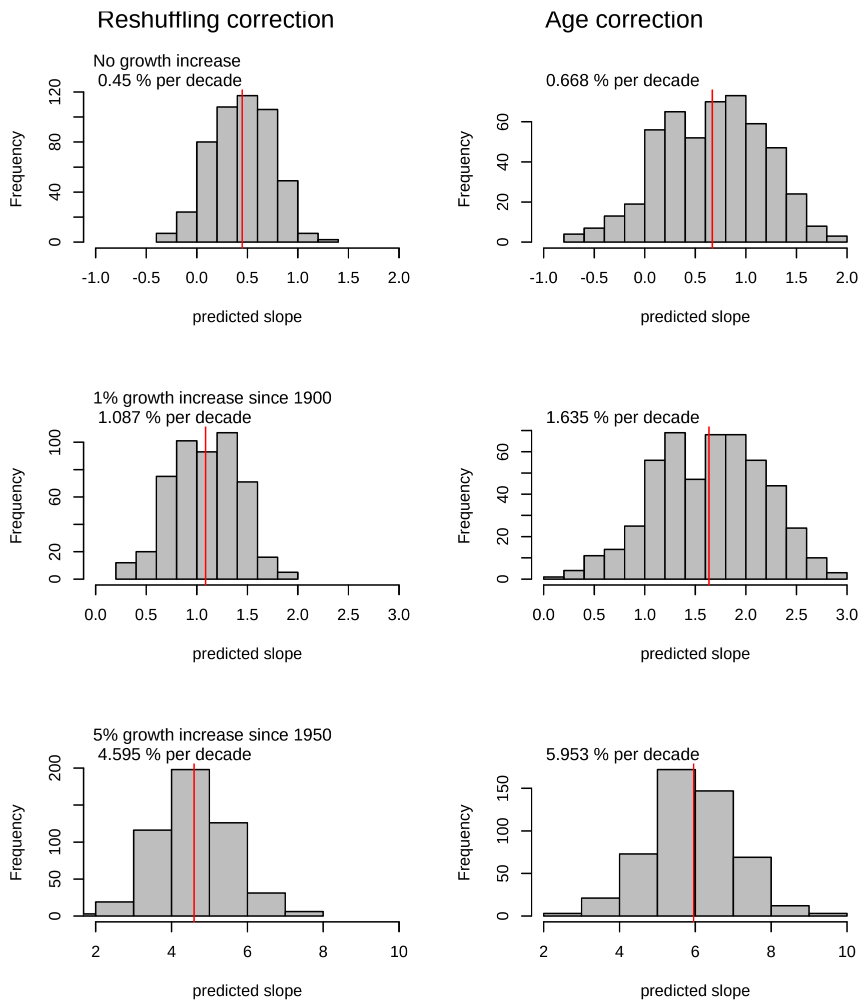

SI Figure 3 Results of tests of two different correction methods for uneven population structures. Histograms show the predicted growth trends for 500 simulated tree populations with uneven recruitment patterns. Left panels show the outcome of the reshuffling approach to correct for the uneven age structure using, and right panels show the outcome of the statistical age correction, where we added age at sample size as additional predictor for growth in the linear model (see methods). The underlying age structure used is the normal age distribution shown in main article Figure 1 with a recruitment centered around 1900 and a standard deviation of 20 years. Three different scenarios were tested forcing growth trend without any growth stimulation (upper panels), with a 1% growth stimulation since 1900 (middle panels), and a 5% growth stimulation since 1950 (lower panels). Note that apparent overestimation of (corrected) growth increase (even when no growth increase is simulated, see upper panels) is due to the slow-growers survivorship bias (cf. Brienen et al. 2012), which results in a very similar effect of 0.68% of spurious growth increases.

Canopy trees, 27 cm

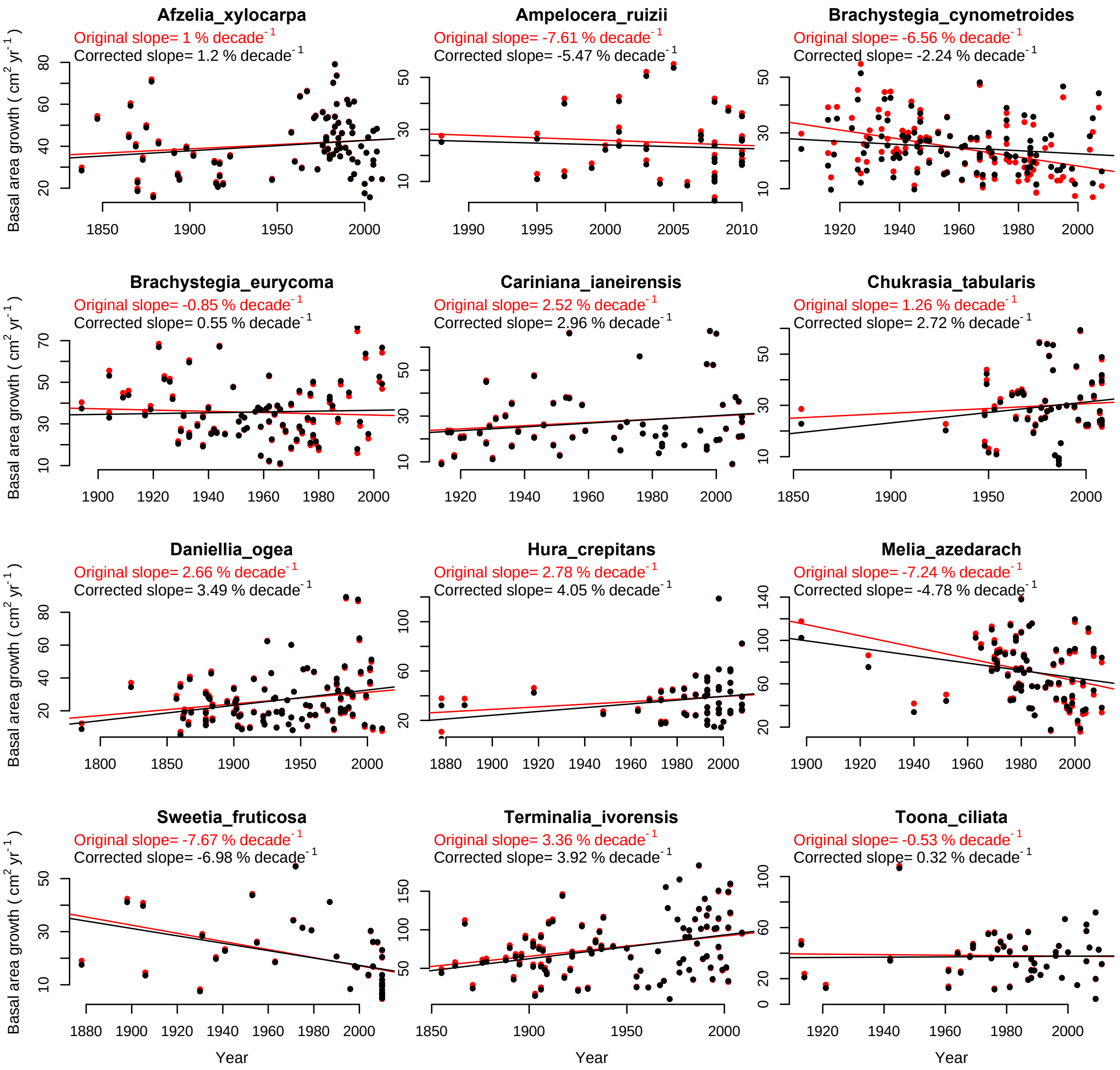

SI Fig. 4 Outcome of the reshuffling correction of growth data for each of the species at 27 cm and 8 cm. Red dots are original growth points, and black dots are corrected growth points. Lines are estimated linear trends. Note that while changes are small in nearly all cases correction of the growth trends resulted in upward adjustments of the observed growth trends.

Understory trees, 8 cm

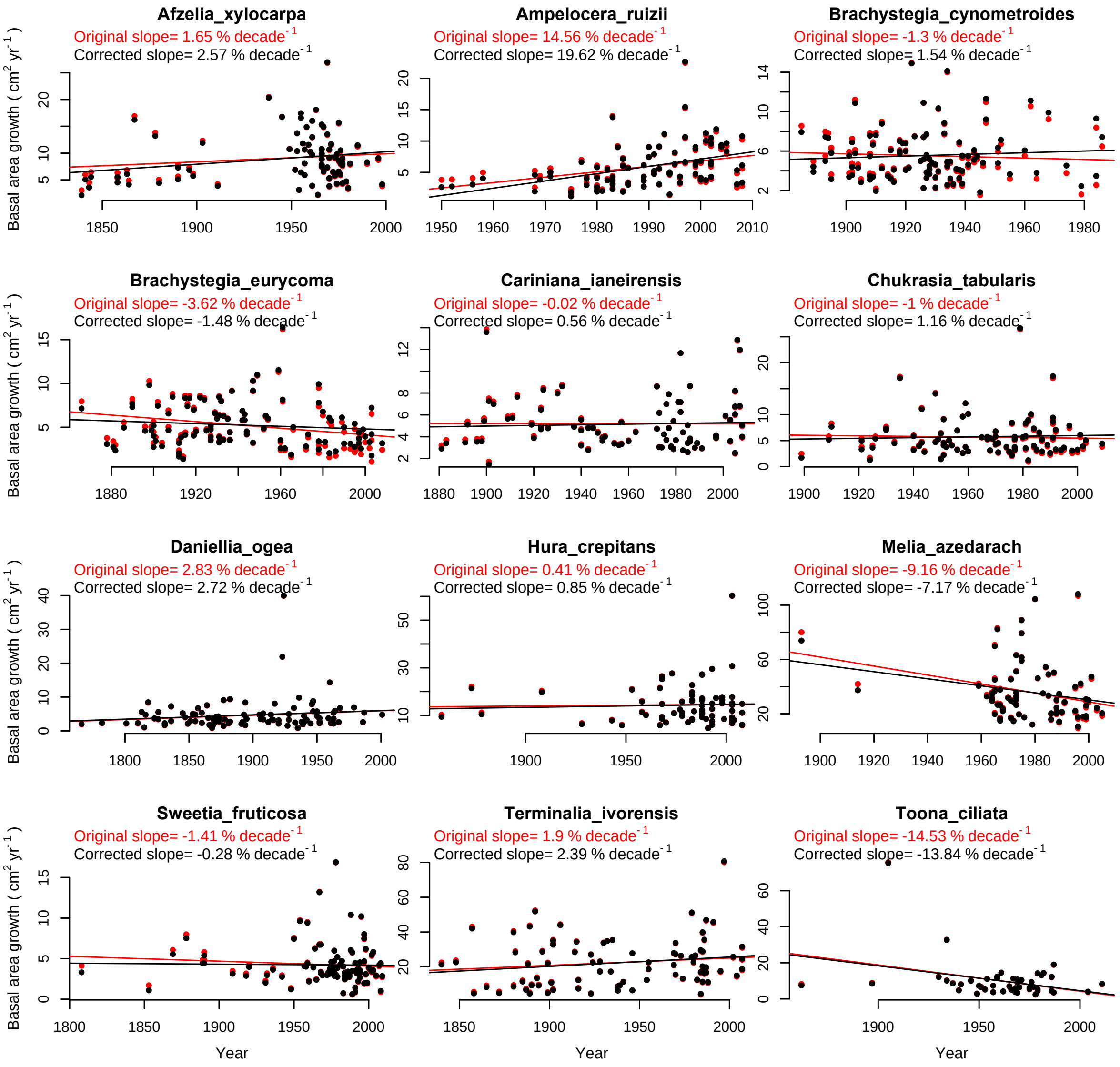

SI Fig. 4 (continued)
